# Morphological change and differential proteomics analysis of gill in *Mytilus coruscus* under starvation

**DOI:** 10.3389/fphys.2023.1150521

**Published:** 2023-03-30

**Authors:** Ze-Wei Liang, Si-Yuan Li, Xiao-Lin Zhang, Chuan-Yue Chen, Wen-Jing Sun, Zhong-Qi Gu, Ji Huang, Jian-Yu He, Peng-Zhi Qi, Bao-Ying Guo, Zhi Liao, Xiao-Jun Yan

**Affiliations:** ^1^ Laboratory of Marine Biology Protein Engineering, Marine Science and Technical College, Zhejiang Ocean University, Zhoushan, Zhejiang, China; ^2^ College of Marine Sciences, Ningbo University, Ningbo, Zhejiang, China; ^3^ China Bureau of Science and Technology Shengsi, Zhoushan, Zhejiang, China; ^4^ Donghai Laboratory, Zhoushan, Zhejiang, China

**Keywords:** *Mytilus coruscus*, starvation stress, gill morphology, differential proteomics, phagocytosis

## Abstract

*Mytilus coruscus* is a dominant shellfish in the Yangtze estuary and its adjacent sea area. Food deprivation often occurs during their growth due to fluctuations in algal abundance caused by seasonal freshwater flushing and high-density aquaculture mode. To investigate the coping strategies of *M. coruscus* to starvation stress, electron microscopy and differential proteomic analysis were performed on the critical feeding organ gill of the mussels after 9 days of starvation. The electron microscopy results showed that the cilia of the mussel gills were dissolved, and the gaps between gill filaments widened under starvation. Differential proteomic analysis revealed that phagocytosis-related proteins such as ATPeV1E, ATPeV1C, LAMP1_2 and CTSL were significantly upregulated, and the phagocytosis pathway was significantly enriched (*p* < 0.05). In addition, the corin content in gill and myeloperoxidase level as well as the number of dead cells in blood were both significantly increased (*p* < 0.05). What’s more, proteomic data suggested that immune maintenance, cellular transport and metabolism related pathways were significantly enriched, which illustrated an immune and metabolism responses under starvation. This study reveals for the first time that phagocytosis functions as an essential strategy for *M. coruscus* to cope with starvation, which provides new scientific knowledge and a theoretical basis for understanding the adaptation mechanisms of mussel to starvation and for rational optimization of mussel culture patterns.

## 1 Introduction

*M. coruscus* is mainly distributed in the northwest Pacific, the Yellow Sea, the Bohai Sea and the East Sea. The coastal mussel resources are largest in Zhejiang province of China. *M. coruscus* are the most important economic species in Zhejiang because of their high nutritional value, low disease and ease of artificial culture. There are more than 14,000 square meters of *M. coruscus* cultivation in the sea area of Shengsi, Zhejiang Province, with a production of 209,800 tons. The mussel cultivation has become the mainstay industry in the country (China Fishery Statistical Yearbook, 2022). However, due to the change of seasons and high-density cultivation mode, the food distribution in the sea area is inhomogeneous, sometimes even insufficient, thus leading to the decreased condition factor of mussel and the delay of harvest time. *M. coruscus* is a typical filter-feeding organism, algae and organic particles in the water column are their natural food sources (Zhang et al., 2022). During their growth, food abundance is crucial for the growth and development (Strohmeier et al., 2009). Starvation stress is a frequent environmental stressor in mussel growth and is associated with cultivation patterns (no artificial feeding), intraspecific competition, the unequal seasonal and spatial distribution of food and environmental changes (Bayne, 1973; Xie et al., 2022). In addition, in our previously study, we found that *M. coruscus* were tolerant to starvation, and no mortality occurred after 9 days of starvation (Xie et al., 2022). According to literature, most bivalves have a strong tolerance to starvation, such as *Pinctada martensi* could survival at least 12 days under starvation (He et al., 2010), and *Lamellidens marginalis* can tolerate 32 days starvation and adapted to maintain a stable immune profile during starvation (Mahapatra et al., 2017). Although bivalves such as mussels have strong starvation tolerance, the underlying responses and adaptation mechanism remains unclear.

Gill is an important feeding organ in mussel (Bracken et al., 2012), the oscillation of various cilia on gills helps mussel to intercept food particles from water column (Rosa et al., 2021), and then selectively ingest them through specific proteins in the mucus such as lectins (Espinosa and Allam, 2018). Particles that cannot be ingested are wrapped in mucus and excreted as pseudofeces (Sonier et al., 2020). Since gills are important filter-feeding organs, we speculate that the temporarily unused filter-feeding function of gills under starvation must lead to some morphological changes. So, to illustrate the morphological changes of gills under starvation, scanning electron microscopy was used to observe the microscopic surface structure of gill tissue.

Differential proteomics analysis can reveal the course and nature of cellular physiological and pathological state, responsive pathways to external environmental stimuli and can help screen for related target proteins (Beltrán et al., 2021). To elucidate the protein expression regulation mechanisms of *M. coruscus* in starvation tolerance, we employed quantitative proteomics to identify the differentially expressed proteins and the related pathways. Phagocytosis is generally considered to have the innate immune functions in cells against foreign infectious sources. However, in addition to the immune function, it was found that phagocytosis is a means to capture external organic particles (Yousefi and Simon, 2009), and it also participates in metabolism and nutrition supply. In most bivalves, blood granulocytes are the most phagocytically active cells (Pila et al., 2016), which undertake the most phagocytosis processes. So, the cell phagocytic activity was usually measured by myeloperoxidase (MPO) enzyme activity, which functions as one of the crucial enzymes for phagocytosis by converting chlorine oxides and hydrogen peroxide to hypochlorite in phagolysosomes (Baldissera et al., 2017). Without of a food source, phagocytosis occurs to recycle energy and nutrients and to remove damaged cells, which may be caused by starvation-induced autophagy, apoptosis, etc. However, the activation of phagocytosis pathway in starvation remains to be further studied.

In the present study, the protein regulatory strategies for mussels to cope with starvation was investigated, and the gill physiological response and various protein expression level as well as their regulatory pathways were identified. The results in the present study reveals for the first time that phagocytosis functions as an essential pathway in mussel to cope with starvation stress, which offers new scientific insights and a theoretical framework for comprehending mussel adaption processes to starvation as well as for rational optimizing mussel culture patterns.

## 2 Materials and methods

### 2.1 Starvation treatment and sample collection

*M. coruscus* were purchased from Shengsi County, Zhoushan City, Zhejiang Province, with an average shell length of 63.81 ± 2.41 mm and an average weight of 24.17 ± 1.69 g (*n* = 50). They were temporarily cultured in a pond with a controlled seawater salinity of 25 and a temperature of 22°C ± 2°C. The pond was continuously aerated and fed with 4.0 × 10^5^ cells/mL of spirulina every day, and the water was changed by one-third after feeding. The mussels were kept in the laboratory for 7 days, followed by the experiment.

36 *M. coruscus* were randomly selected and divided into two groups, which were recorded as the control group and the experimental group. The control group was regularly fed with commercial spirulina pollen at a concentration of 4.0 × 10^5^ cells/mL daily, and half of the water was changed after each feeding. In contrast, the experimental group was not fed, and only the water was changed. After 9 days of starvation, gill tissues were dissected from the feeding and starved groups, homogenized, and centrifuged (4°C, 3,000 × *g*, 10 min), and the supernatant was used for subsequent experiments.

### 2.2 Scanning electron microscope observation

The middle segment of the external gill was taken, washed with sterile seawater and fixed with 2.5% glutaraldehyde at 4°C for 12 h. After fixation, the samples were rinsed with 0.1 M pH7.0 phosphate buffer and then fixed with 1% osmium acid for 1–2 h. The samples were then dehydrated with ethanol solutions of graded concentrations (30%, 50%, 70%, 80%, 90% and 95%), each concentration for 15 min, followed by two treatments with 100% ethanol for 20 min each. The samples were treated with a mixture of ethanol and isoamyl acetate (V: V = 1: 1) for 30 min and treated with pure isoamyl acetate for 1 h or left overnight. After critical point drying, the samples were coated with gold spray by an ion sputterer (ION SPUTTER JFC-1100). The treated samples were observed in a scanning electron microscope (SU8010, Hitachi, Inc.) and photographed.

### 2.3 Differential proteome sequencing and analysis

Total gill tissue protein was extracted using the Total Tissue Protein Extraction Kit (Beijing Bangfei, AP0601-50) in combination with Complete Protease Inhibitor EASY packs (Roche). The total protein concentration of each sample was determined by the Micro BCA Protein Assay Kit (Roche), and the integrity of the samples was detected by 10% polyacrylamide gel electrophoresis. Two protein samples from each group were mixed equally as one biological replicate. Finally, three biological replicates were obtained for each group, and a total of six samples from the two groups were sent to Shanghai Yuansheng Biomedical Technology Co., for proteome sequencing.

For each sample, 60 μg of protein solution was added to 5 μL of 1 M DTT solution and mixed well at 37°C for 1 h. 20 μL of 1 M IAA solution was added, mixed and reacted at room temperature and protected from light for 1 h. All samples were added to ultrafiltration tubes and centrifuged, and the collection solution was discarded. 100 μL of UA (8 M urea, 100 mM Tris-HCl, pH 8.0) was added to ultrafiltration tubes, centrifuged, and the collection solution was discarded and repeated twice. 100 μL of 50 mM NH_4_HCO_3_ were added, centrifuged and the collection solution were discarded again, repeat this step three times and renew the collection tube. After that, trypsin (Promega) was added to the ultrafiltration tube at a 50:1 ratio of protein to the enzyme and digested at 37°C for 12–16 h.

A nanoliter flow rate HPLC liquid phase system EASY nLC/Ultimate 3,000 chromatograph (Thermo Scientific) was used to separate the enzymatically digested protein samples. The mobile phase A solution was 0.1% formic acid aqueous solution, and the mobile phase B solution was 0.1% formic acid acetonitrile aqueous solution (80% acetonitrile). The samples were loaded by an autosampler onto a mass spectrometry pre-column (PepMap 100, nanoViper C18,100 Å, 3 μm, 100 μm × 20 mm) and then separated by an analytical column (PepMap 100, nanoViper C18,100 Å, 1.9 μm, 150 μm × 120 mm). Liquid phase gradient was set as follows: 0–10 min, 0%–6% for B; 10–15 min, 6%–10% for B; 15–70 min, 10%–30% for B; 70–80 min, 30%–40% for B; 80–85 min, 95% for B. Each sample was separated by capillary high-performance liquid chromatography and analyzed by mass spectrometry using a Q Exactive HF-X mass spectrometer (Thermo Scientific). The specific parameters were set as full scan primary spectrum range 350–1,550 m/z, resolution 120,000, maximum integration time 20 ms, 3 × 106 ion targets, secondary spectrum range 200–2000 (m/z), resolution 15,000, maximum integration time 30 ms, 2 × 104 ion targets.

MaxQuant software (version 1.6.17) was used for protein comparison and identification of mass spectrometry data, and the search database was the *M. coruscus* genome data (SRA database number: PRJNA635756). The normalized protein quantification data were set to a significance threshold of 0.01 for the False Discovery Rate (FDR), and the regular *M. coruscus* group was used as a control to obtain the fold change (FC), and FC ≥ 1.5 or FC ≤ 0.66 (*p* < 0.05) were defined as significantly differentially expressed proteins.

GO (Gene ontology) and KEGG (Kyoto Encyclopedia of Genes and Genomes, KEGG) functional annotation and enrichment analysis were performed on the identified differential proteins. All differential proteins were first mapped against each term of the GO database, the number of proteins identified in each term was calculated, and then hypergeometric tests were applied to identify GO entries that were significantly enriched in differential proteins compared to all protein backgrounds. The KEGG pathway significant enrichment analysis was performed using the KEGG automatic annotation server (KAAS) software. The KEGG metabolic pathway significant enrichment analysis was performed the same way as the GO functional enrichment analysis. The most essential biochemical metabolic and signal transduction pathways involved in the differential proteins were identified.

### 2.4 Real-time quantitative PCR (RT-qPCR) validation

The total RNA of the samples was extracted using an RNA extraction kit (EasyPure^®^ RNA Kit, TransGen) and verified by reverse transcription with a reverse transcription kit (PrimeScript™ RT reagent Kit, TaKaRa) followed by RT-qPCR. The selected genes and corresponding encoded proteins are shown in Table 1. β-actin was used as the internal reference gene, the RT-qPCR amplification was taken in three replicates, and the data were processed by the 2^−△△Ct^ method.

**TABLE 1:** Differentially expressed proteins and primer sequences used in RT-qPCR.

Protein IDs	Description	Expression change	Primer sequences (5′-3′)	Tm value (°C)
A0A6J8D0E4	HIF1AN	Down	F: CAC​CTT​CTC​CAC​CGA​AGT​CAT​TCC	60.09
			R: TAA​GTT​CCA​ATG​CCT​TGC​TGA​TGC	58.81
A0A6J8DZK5	DeoA	Down	F: GAG​CCA​GGA​GCG​GAT​ATG​TTC​AAG	60.20
			R: TGC​ACC​AAG​TCT​ACC​ACT​GAT​TTC​G	59.20
A0A6J8EEC3	Plasminogen	Down	F: GCA​CAG​TTG​GTG​ACA​CAC​ATT​ACA​G	58.53
			R: CCG​CCT​TAT​TGA​TGA​CGA​CGA​CAG	60.36
A0A6J8BS55	SEPT4	Down	F: TTC​CCA​GAA​TGT​GAC​AGC​GAT​GAA​G	59.28
			R: TCT​TCC​CTC​CTG​CCT​CTA​CAA​CTG	60.60
A0A6J8CQU3	PepP	Down	F: AAC​CCA​CCT​GAG​CGA​TGT​AAA​ACT​G	59.48
			R: GAT​AGT​CAG​CAC​GGT​CTG​GAA​CAT​G	59.80
A0A6J8CJ45	CORIN	Up	F: CAC​GAA​CAG​CAA​CTA​TGA​CCT​CTC​C	59.74
			R: CTG​ACA​CGC​CTC​CTT​CCA​TGT​TG	60.99
A0A6J8AGJ9	SDCBP	Up	F: GAC​AGT​GCA​GGG​TAC​ATT​GGT​TTT​G	58.68
			R: AAC​ACA​CCA​TTT​CTT​GCT​GCT​GAA​G	58.10
A0A6J8CA19	CTSL	Up	F: AAG​TTC​CTG​CCT​CCT​CTC​AAT​GTT​G	59.17
			R: ACA​TGA​ACC​ACA​CTG​TCC​CTG​ATT​C	59.14
A0A6J8EAW4	MMP17	Up	F: ACA​CAA​GAC​AGC​TTG​GAG​GTT​CAT​C	59.27
			R: CTG​CGT​CGC​TTC​CAC​CAA​CTT​C	61.82
A0A6J8C029	COL22A	Up	F: GAG​CCT​GTG​ACA​TTC​CAG​CCA​ATA​G	60.18
			R: CAA​TAC​TGT​CAG​TTC​GCT​CCC​TTC​C	59.82

### 2.5 Corin protease content and MPO enzyme activity assay

To verify the accuracy of the proteomic data, the content of corin protease which is significantly upregulated was measured. The total protein of the control and starved gill tissues was extracted according to the method in above mentioned, and different concentrations of standards were prepared according to the Mannose Binding Lectin-Associated Serine Protease 1 (MASP1) ELISA Kit (Shanghai Enzyme-linked). Blank wells (no sample and enzyme reagents were added to the blank control wells, and the rest of the steps were the same), standard wells and wells for the samples to be tested were set up on the enzyme plate. 10 μL of each sample was used to be tested (the final dilution of the sample was 5 times). After 30 min of incubation at 37°C, 50 μL of enzyme standard reagent was added to each well after washing, and color development was performed by adding color developer. The standard concentration was used as the horizontal coordinate, and the OD value was used as the vertical coordinate to draw the standard curve. The corresponding concentration was found from the standard curve according to the OD value of the sample and then multiplied by the dilution, which was the actual content of corin protease. Mussel blood was taken from the starved and control groups and MPO activity was determined strictly according to the procedures of the Myeloperoxidase (MPO) Test Kit (Nanjing Jiancheng).

### 2.6 Identification of cell viability in blood

Mussel blood from the starved and control groups was stained with 4% Trypan Blue and observed under a microscope. Cell concentration and cell viability in the blood were calculated using a cell counter (LUNA Ⅱ, Korea).

### 2.7 Data analyses

All data were analyzed statistically by one-way analysis of variance (ANOVA) using SPSS software package. All data were presented as mean ± standard deviations (SD), and a significant difference was shown as *p* < 0.05.

## 3 Results

### 3.1 Morphological change of gill

**FIGURE 1:**
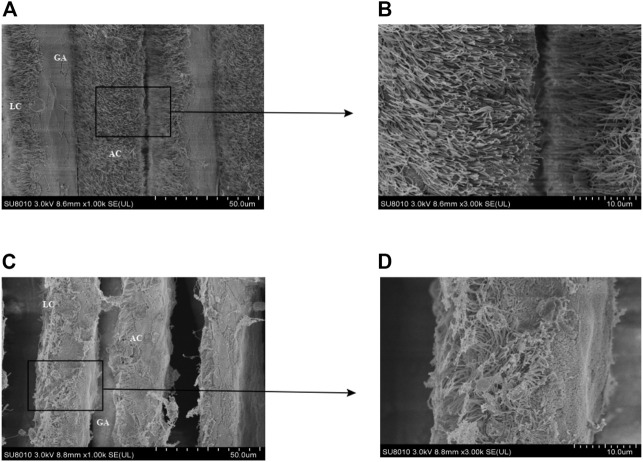
SEM analysis of *M. coruscus.* **(A)** control group; **(B)** black enlarged area in Figure **(A)**; **(C)** starvation group; **(D)** black enlarged area in Figure **(C)**; GA, gill axis; LC, lateral cilia; AC, terminal cilia.

The *M. coruscus* gill tissue includes two parts, gill filaments and gill shafts, in which gill filaments are attached to the gill shafts vertically, and there are a large number of cilia distributed on the lateral side of the gill filaments, which can be divided into lateral cilia and terminal cilia depending on the location. In the control group of the *M. coruscus*, the primary gill filaments were solid and compact, with well-developed and dense cilia distributed on the lateral surface (Figures 1A, B). However, the gills of the starved group showed obvious changes in the surface structure of the primary gill filaments, and the lateral cilia showed strong local autolysis. In addition, the distribution of terminal cilia was disturbed and the top of the cilia showed a phenomenon similar to lysis caused by structural disruption (Figures 1C, D).

### 3.2 Proteomic data quality assessment

The quality of the proteomics data was judged based on the length of the peptides identified by mass spectrometry. The length of the peptides identified by mass spectrometry peaked between 10 and 13 AA, and 90% of the peptides were within 24 AA, which was credible (Supplementary Figure S1). The distribution of the number of identified peptides corresponding to the identified proteins showed that about 50.01% of the modified peptides scored above 81.77. The median peptide score was 81.77, indicating that the mass spectrometry detection quality was good, the identification and analysis results were reliable, and the obtained data could be used for subsequent analysis. The mass spectrometry proteomics data have been deposited to the ProteomeXchange Consortium (http://proteomecentral.proteomexchange.org) *via* the iProX partner repository with the dataset identifier PXD040908.

### 3.3 Identification and screening of differentially expressed proteins

**FIGURE 2:**
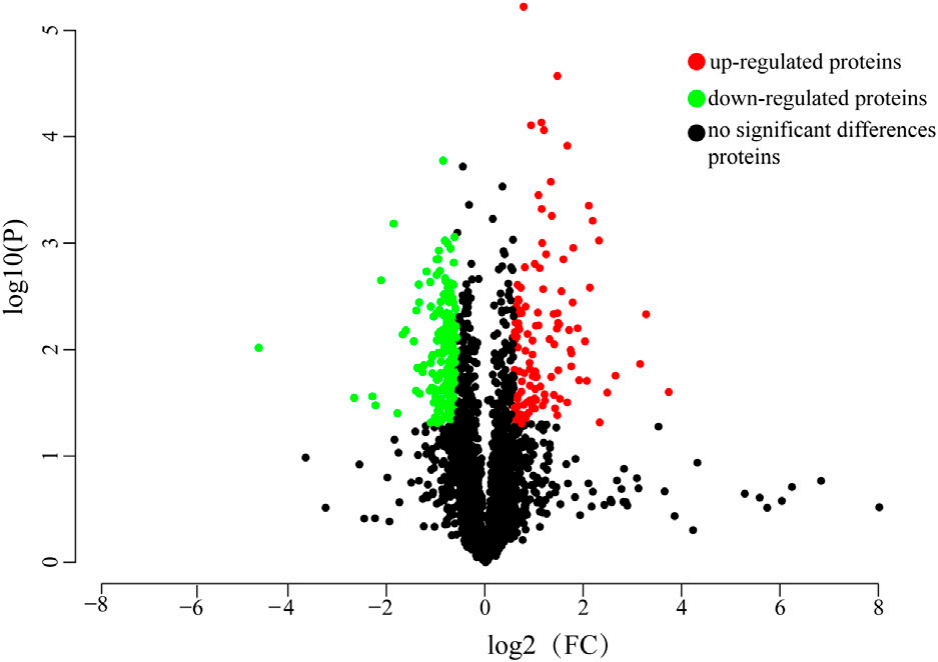
Volcano plot of differentially expressed proteins. Green dots represent downregulated proteins, red dots represent upregulated proteins, black dots represent genes with no significant differences in expression.

Proteome Discoverer software v2.5 was used for raw data retrieval. The qualitative and quantitative protein data were obtained by comparing the raw mass spectrometry data of the two sample groups using the Label-free quantitative analysis method. The sample quantitative ratio distribution showed that the sample protein ratio was close to 1, which ensured the comparability between groups and the quantification accuracy during quantitative analysis. A total of 19,560 unique peptides were identified, and 2,780 proteins were identified, of which 2,715 protein quantification data were used for subsequent proteomic differential analysis after quality control. A total of 319 differentially expressed proteins were identified in the gill tissues of control and starvation groups as screened by the fold difference (FC > 1.5 or FC < 0.66) and significance level (*p* < 0.05). Among them, 116 proteins were significantly upregulated, and 203 were significantly downregulated after starvation. The number of differentially expressed proteins is shown in Figure 2.

Further, 21 significantly upregulated expressed proteins were identified using a 3-fold or more change in protein abundance as a screening criterion, among which 16 proteins had functional annotations (Table 2), namely, CORIN (transmembrane serine protease, A0A6J8CJ45), SDCBP (syntenin-1, A0A6J8AGJ9), MMP17 (matrix metalloproteinase-17, A0A6J8EAW4), MMP14 (matrix metalloproteinase-14, A0A6J8CKJ2), CTSL (cathepsin L, A0A6J8CA19), LAMP1_2 (lysosomal-associated membrane protein 1/2, A0A6J8EL29), PLD3_4 (phospholipase D3/4, A0A6J8AMK0), ATPeV1C (V-type H^+^-transporting ATPase subunit C, A0A6J8AKM6), PXDN1 (peroxidase-1, A0A6J8BTI4), PXDN2 (peroxidase-2, A0A6J8BSZ8), FTH1 (ferritin heavy chain, A0A6J8AL47), COL22A (collagen type XXII alpha, A0A6J8C029), ATPeV0A (V-type H^+^-transporting ATPase subunit a, A0A6J8D4V9), SPG20 (spartin, A0A6J8BCA7) and ABCB1 (ATP-binding cassette, subfamily B (MDR/TAP), member 1, A0A6J8D457). Another nine significantly downregulated proteins were identified, six of which had annotation results (Table 3), in the order of DeoA (thymidine phosphorylase, A0A6J8DZK5), HGF (hepatocyte growth factor, A0A6J8EEC3), HIF1AN (hypoxia-inducible factor 1-alpha inhibitor, A0A6J8D0E4), SEPT4 (saptin 4, A0A6J8BS55), CD109 (CD109 antigen, A0A6J8AXQ8) and PepP (Xaa-Pro aminopeptidase, A0A6J8CQU3).

**TABLE 2:** Significant upregulated proteins.

Accession No.	Annotation	Mol. weight (kDa)	Sequence coverage (%)	Unique peptides	Score
A0A6J8CJ45	CORIN	269.62	6.5	12	43.668
A0A6J8BQR5	Uncharacterized protein	26.981	77.2	18	97.935
A0A6J8C029	COL22A	107.58	21.4	17	54.385
A0A6J8D250	Uncharacterized protein	82.28	2.8	2	2.467
A0A6J8AGJ9	SDCBP	24.681	23.8	5	11.627
A0A6J8EAW444	MMP17	24.148	22.1	6	14.365
A0A6J8EA38	Uncharacterized protein	83.546	8.9	5	19.476
A0A6J8CA19	CTSL	35.765	19.9	5	36.23
A0A6J8EL29	LAMP1_2	56.261	20.7	9	28.608
A0A6J8EJW6	Uncharacterized protein	54.57	18.9	8	62.763
A0A6J8CQS0	Uncharacterized protein	156.37	36.8	33	323.31
A0A6J8AMK0	PLD3_4	53.322	7.3	3	4.2693
A0A6J8CKJ2	MMP14	56.473	26.5	13	32.527
A0A6J8AKM6	ATPeV1C	45.803	19.6	8	14.097
A0A6J8BTI4	PXDN1	28.864	29.3	5	35.648
A0A6J8BSZ8	PXDN2	23.404	16.3	3	7.7963
A0A6J8AL47	FTH1	19.723	25.7	4	36.559
A0A6J8BCA7	SPG20	48.008	6.9	3	11.15
A0A6J8D457	ABCB1	88.466	28.2	14	29.393
A0A6J8D4V9	ATPeV0A	74.216	11.1	6	10.739
A0A6J8BIN1	COL6A	49.639	36.2	16	78.961

**TABLE 3:** Significant downregulated proteins.

Accession No.	Annotation	Mol. weight (kDa)	Sequence coverage (%)	Unique peptides	Score
A0A6J8DZK5	DeoA	48.748	26	11	27.564
A0A6J8EEC3	HGF	107.23	28	22	143.58
A0A6J8BFT9	Uncharacterized protein	20.389	28.6	4	22.246
A0A6J8D0E4	HIF1AN	38.717	21.5	5	20.25
A0A6J8BS55	SEPT4	94.288	7.7	5	8.7635
A0A6J8AXQ8	CD109	62.985	14.2	7	15.623
A0A6J8CQU3	PepP	82.049	8	5	13.013
A0A6J8BGV1	Uncharacterized protein	72.042	15.3	8	28.175

### 3.4 GO and KEGG annotation analysis of differential expressed proteins

**FIGURE 3:**
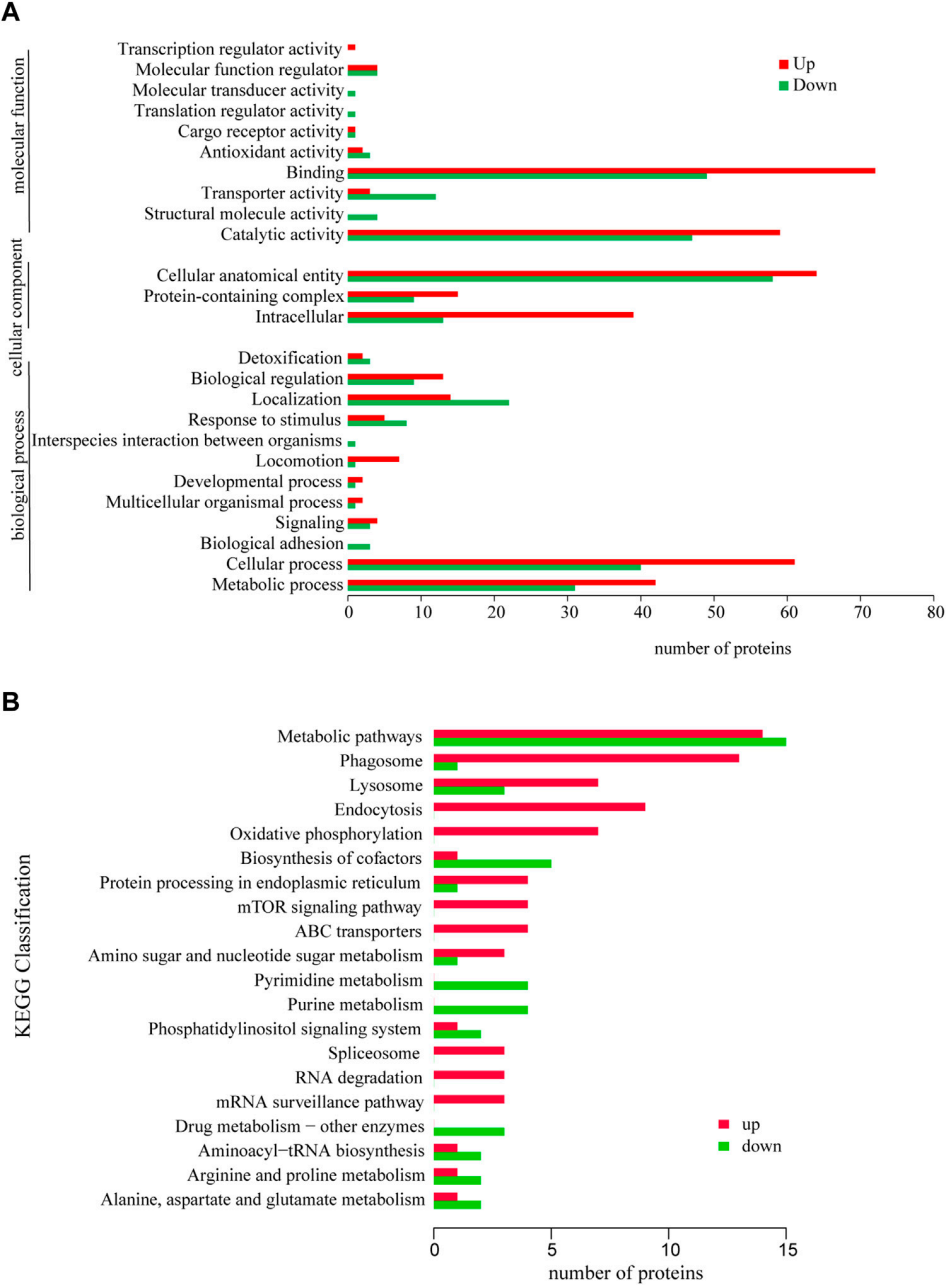
GO annotation analysis of differentially expressed proteins **(A)** and KEGG annotation analysis of differentially expressed proteins **(B)**. *p < 0.05 or **p < 0.01 versus starvation group.

The GO functional annotation results showed that the differentially expressed proteins were involved in 12 biological processes, including Cellular process (GO: 0009987), Localization (GO: 0051179) and Metabolic process (GO. 0008152), Cellular anatomical entity (GO: 0110165), Intracellular (GO: 0005622) and Protein-containing complex (GO: 0032991), the molecular functions are mainly Binding (GO: 0005488) and Catalytic activity (GO: 0003824) (Figure 3A).

The annotation analysis of KEGG metabolic pathways showed that the differentially expressed proteins were mainly involved in Metabolic pathways (ko01100), Phagosome (ko04145), Endocytosis (ko04144), Lysosome (ko04142), Metabolic pathways (ko01100), Biosynthesis of cofactors (ko01240), Pyrimidine metabolism (ko00240) and Purine metabolism (ko00230) (Figure 3B).

### 3.5 GO and KEGG enrichment analysis of differential expressed proteins

**FIGURE 4:**
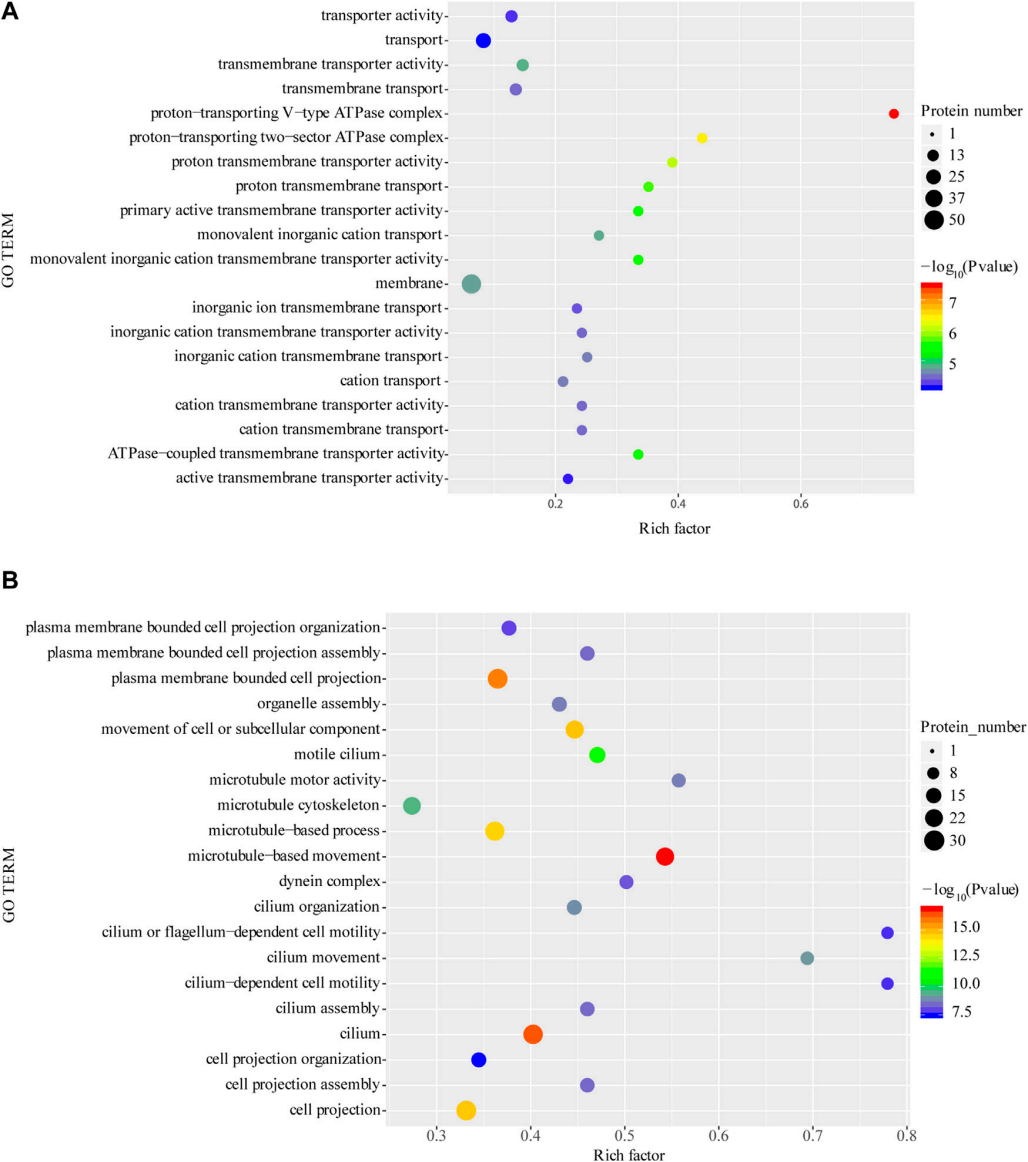
Enrichment of GO function of upregulated proteins **(A)** and downregulated proteins **(B)**. *p < 0.05 or **p < 0.01 versus starvation group.

The GO enrichment of differentially expressed proteins showed that, the upregulated expressed proteins are mainly including V-type proton-transporting ATPase complex, proton-transporting two-region compartment ATPase complex and proton transmembrane transport protein activity (Figure 4A). The top 10 significantly upregulated proteins are serine protease, multiligand glycan-binding protein, matrix metalloproteinase, histone protease, lysosome-associated membrane proteins, phospholipase D, protein phosphatase, peroxisome homologs, heavy peptide ferritin, metal ion binding and ABC transporter protein.

Among the downregulated differentially expressed proteins, cilia- or flagellar-dependent cell motility, ciliary motility, motile ciliated cells and other related functions were significantly enriched (Figure 4B). The top 5 significantly downregulated proteins, including thymidine phosphorylase, hypoxia-inducible factor, and phosphoenolpyruvate phosphodiesterase which involved in catalytic activity, another significantly downregulated proteins SEPT4 and cell surface antigen are involved in cellular carcinogenesis and cellular immunity, respectively.

**FIGURE 5:**
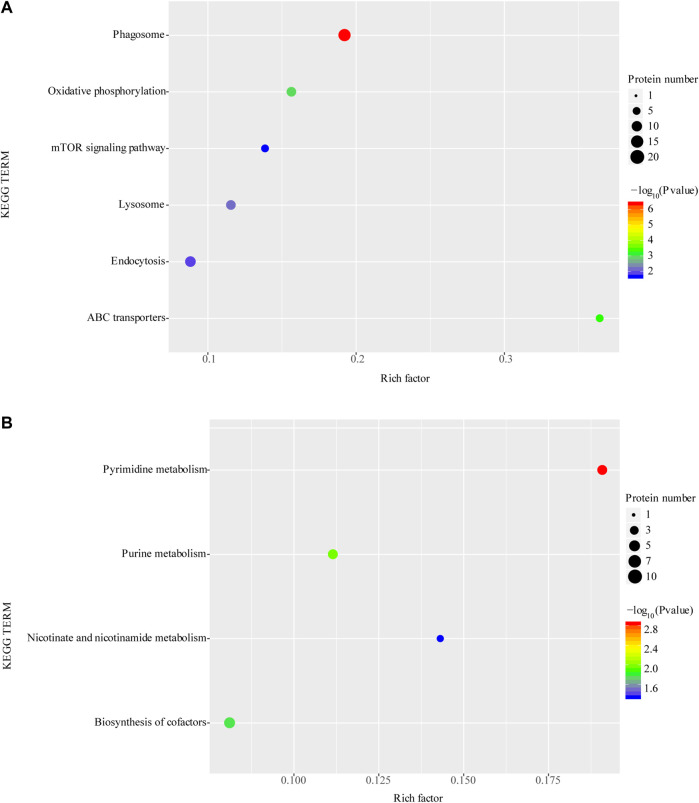
Enrichment of KEGG function of upregulated proteins **(A)** and downregulated proteins **(B)**. *p < 0.05 or **p < 0.01 versus starvation group.

The KEGG enrichment analysis showed that upregulated protein functions were mainly enriched in the phagosome, oxidative phosphorylation, mTOR, lysosome, and endocytosis pathways (Figure 5A). In contrast, downregulated proteins were mainly enriched in pyrimidine metabolism, purine metabolism nicotinic acid and nicotinamide metabolism and cofactor synthesis pathways (Figure 5B).

### 3.6 RT-qPCR validation of differentially expressed proteins

**FIGURE 6:**
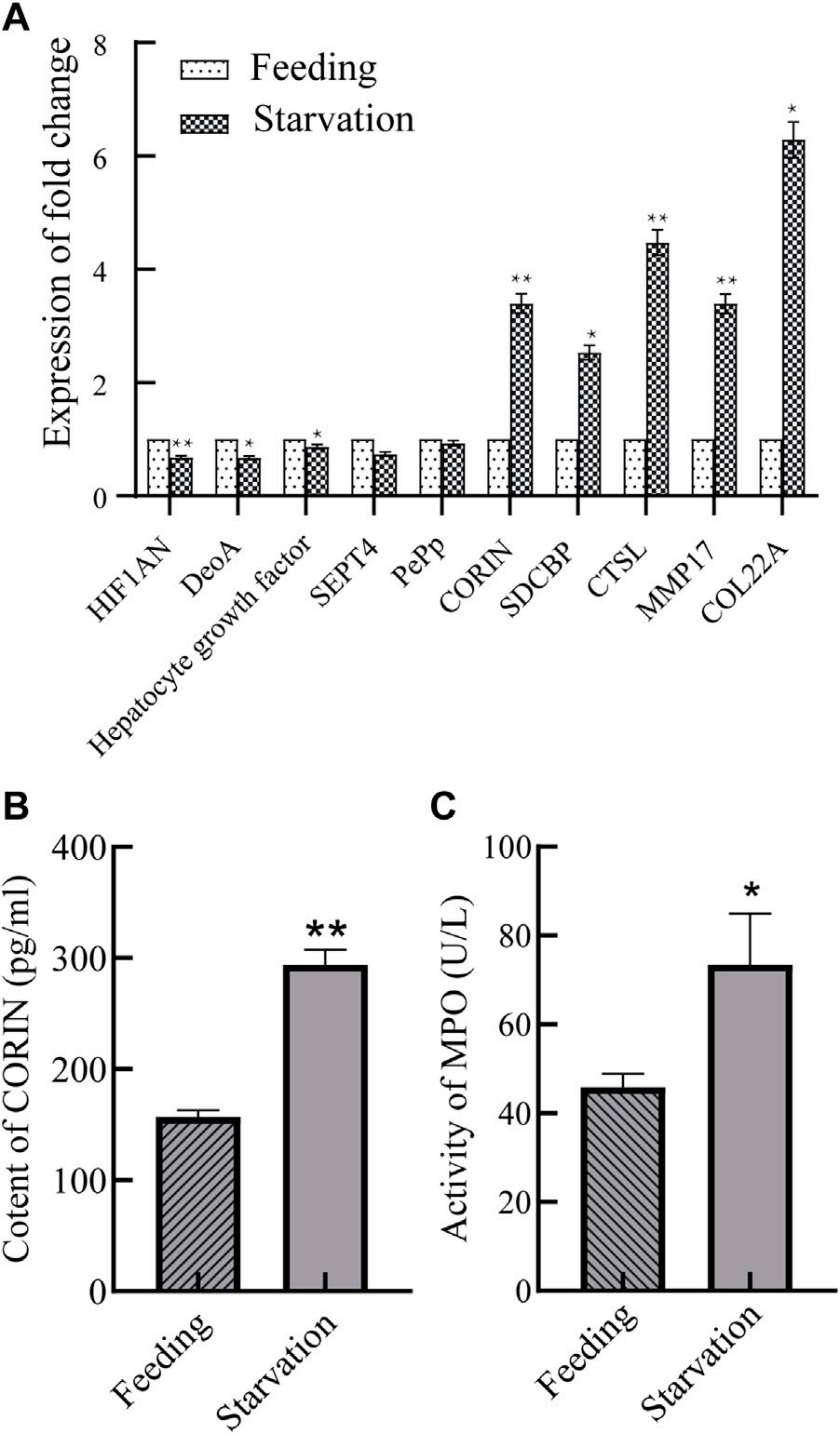
Expression changes of differentially expressed protein-coding genes **(A)** and changes of CORIN content **(B)** and activity of MPO in blood **(C)**. *p < 0.05 or **p < 0.01 versus starvation group.

Differential expressed proteins related to phagocytosis, catalysis and metabolism, etc. (protein numbers and annotations are detailed in Table 1) were selected for RT-qPCR validation of the expression of their coding genes of control and starved groups, and the results are shown in Figure 6A. The expression changes of selected genes were highly consistent with the results in the proteome, thus indicating that the proteome data are reliable.

### 3.7 Corin protease content and MPO enzyme activity

The serine protease corin with significant upregulated expression changes (protein number: A0A6J8CJ45, annotated CORIN) was selected and the changes in the content of this protease in the gill tissue of *M. coruscus* were detected. The results are shown in Figure 6B. The levels of corin protease were 156.7 and 293.9 pg/mL before and after starvation, respectively, and the levels of this protein increased 1.9-fold after starvation. In addition, Figure 6C shows that the MPO enzyme activity in the blood of *M. coruscus* starved for 9 days was approximately 1.62 times higher than that in the control group.

### 3.8 Identification of cell viability in blood

**FIGURE 7:**
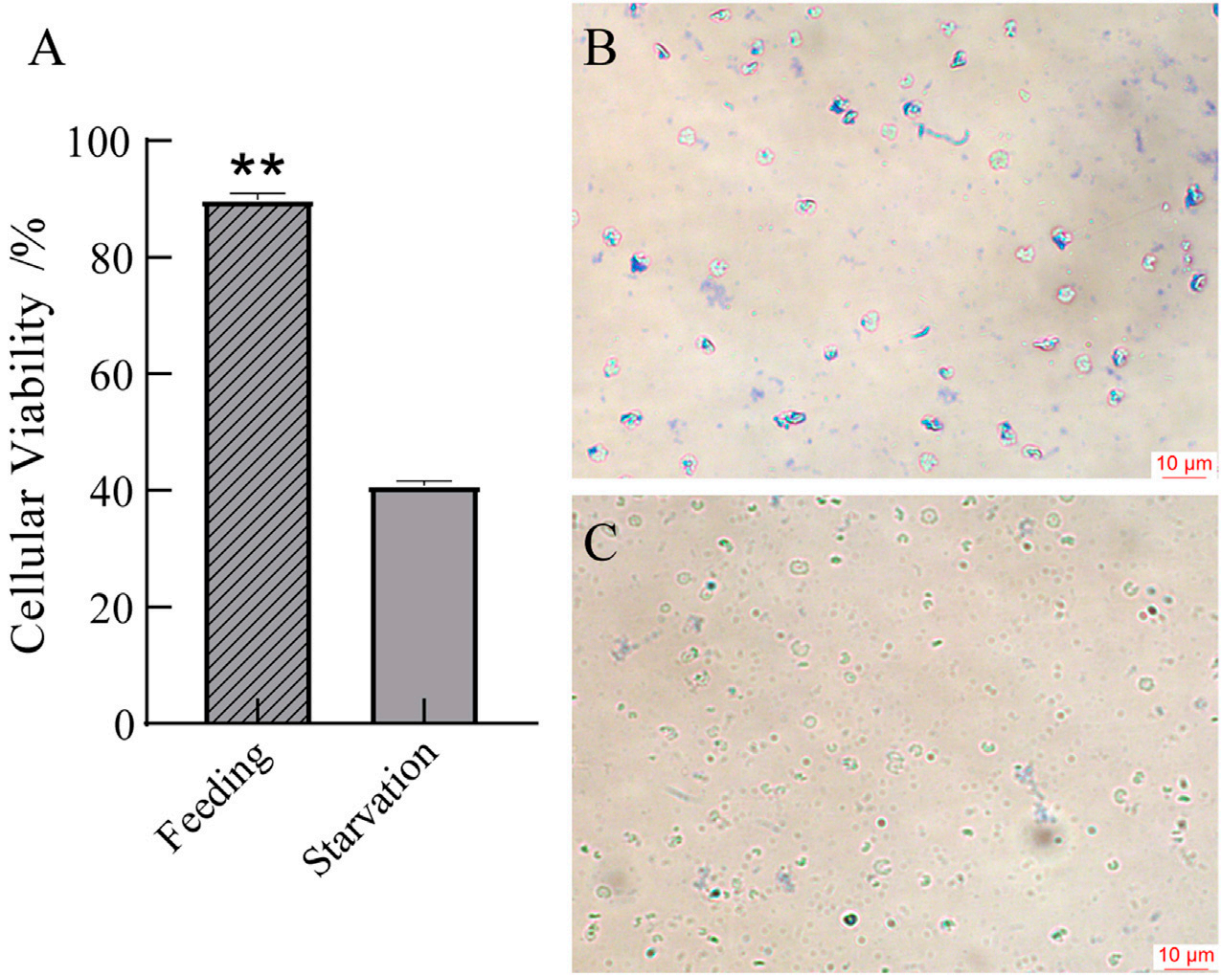
Cellular viability in blood **(A)** and microscopic observation of blood in the feeding group **(B)** and the starvation group **(C)**. *p < 0.05 or **p < 0.01 versus starvation group.

As is shown in Figure 7, the total cell concentration in the starved group was 2.0 ± 0.4 × 10^5^ cells/ml and the active cells accounted for 40.8% ± 0.6% of the total cells, as identified by the Trypan blue staining. However, the cell concentration in the control group was 2.0 ± 0.1 × 10^5^ cells/ml, and the active cells accounted for 89.8% ± 0.1% of the total cells. This indicates that starvation leads to a significantly increase in dead cells in the blood.

## 4 Discussion

In order to investigate the physiological response and protein expression regulation of *M. coruscus* to starvation, we conducted a preliminary study to analyze the morphological change and the differential proteomics in the gill of *M. coruscus*. We found that many gill cilia dissolved under starvation, and the intervals between gill filaments widened compared with the control group. Similar results have been reported in *Charybdis japonica* and *Megalobrama amblycephal* (Sun et al., 2015; Sun et al., 2020). Gill filament oedema, cuticle thinning and partial dissolution of mitochondria were observed in the gill tissue of *C. japonica* after 16 days of starvation. The thickness of the interlamellar matrix of gill lamellae in *M. amblycephal* was also gradually reduced with the extension of starvation. Cilia of bivalve gill tissues play an important role in food uptake, food particles were intercepted and screened by anterior cilia first, and then delivered to the food channel. Researches have been showed that lateral cilia play important roles in the interception resistance of the water flow (Garrido et al., 2012), so when the mussel cilia autolysis softens, the efficiency of the mussel cilia to intercept food particles will be significantly reduced (Jørgensen, 1975). Gill is the most essential tissue in *M. coruscus*, which plays important roles both in feeding and immunity. Since no food feeding during starvation, the gill cilia are shed and the space between gill filaments widen. Those morphology changes are a physiological response to fit starvation. On the other hand, the widen of gill filaments is also conducive to particles accession once the food is available.

**FIGURE 8:**
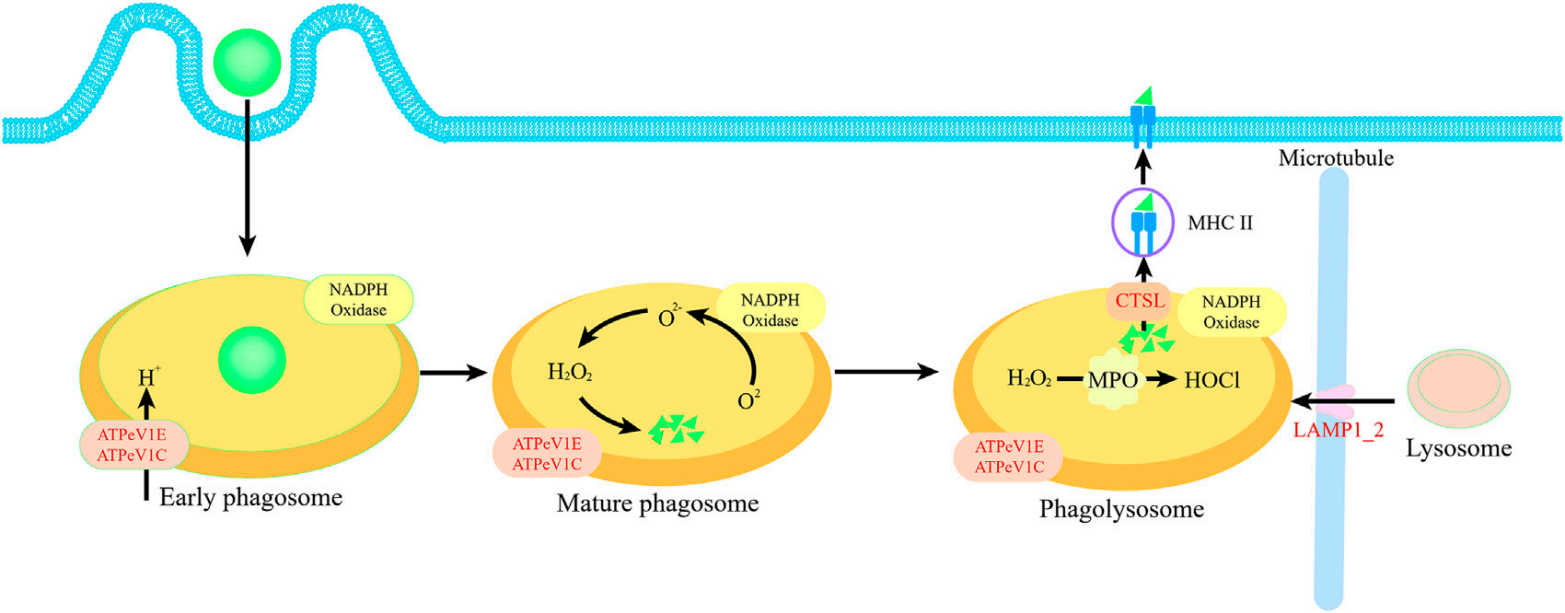
Schematic diagram of phagocytosis pathway. Four proteins ATPeV1E, ATPeV1C, LAMP1_2 and CTSL in red font are significantly upregulated proteins in relation to phagosome pathway.

We further analyzed the proteomics of gill to clarify the protein regulation mechanism. Through the KEGG enrichment analysis, we identified four proteins that significantly upregulated about phagosome pathway, namely, ATPeV1E, ATPeV1C, LAMP1_2 and CTSL. Among them, ATPeV1E and ATPeV1C play a proton transfer role in early phagosomes. LAMP1_2 plays a role in trans microtubule transport of lysosomes to enable mature phagosomes to form phagolysosomes, CTSL can further cleave organic particles in phagolysosomes and enable them to form antigens for distribution to the cell surface (Blander and Medzhitov, 2004) (Figure 8). Phagocytosis is attributed to two factors for mussels. Firstly, it can recycle energy and nutrients from damaged cells without a food source. The presence of a large number of damaged cells in the blood can be demonstrated by trypan blue staining. At the same time, the results of the electron microscopy scan also provide reasonable verification of cell damage in the gill. The function of shellfish to engulf their necrotic cells and supply nutrients has been reported (Mandujano-Tinoco et al., 2021). So, we speculated the intrinsic phagocytosis process under starvation as follows, the cilia cells in the gill were firstly shed and died after the starvation stimulation, and then as the blood cells flow through the gills, the granulocytes in the blood engulf these dead cells in gills and break them down into energy and nutrients to supply to other tissues. In addition, we detected a significant increase in MPO activity, which further validates the occurrence of phagocytosis, as MPO functions to convert chlorine oxides and hydrogen peroxide to hypochlorite in the phagolysosomes and is one of the most essential enzymes for phagocytosis. Secondly, cellular phagocytosis is a central mechanism to defend against inflammation and defense against infection factors. Hampton et al. (1998) found that hypochlorous acid is a mediator of oxygen-dependent bacterial killing in neutrophil phagocytic vesicles and that the level of hypochlorous acid released from phagosomes during phagocytosis is lethal for bacteria. It also ensures that mussel has some resistance to external infection factors even if the cells are damaged during starvation. Phagocytosis against external infection factors also occurs in other bivalves. For example, Lei et al. (2022) found that ctenophore scallops are highly phagocytic against *Escherichia coli* and *Staphylococcus aureus*. Significant upregulation of ATPeV1E, ATPeV1C, ATPeV1H and other proteins can regulate not only phagocytosis but also mTOR pathway. This pathway functions as a key homeostatic regulator of cell growth (Dunlop and Tee, 2014). Unlike phagocytosis, autophagy is a process by which mTORC1 maintains nutrient homeostasis through the process of lysosomal biogenesis and autophagy, which is used by cells to recycle damaged or unwanted organelles and macromolecules. In contrast, phagocytosis is achieved by engulfing external organic particles into phagocytes by cytokinesis and then lysing them with phagolysosomes. Although autophagy has been shown to respond to starvation and to accelerate recovery from cellular damage (Moore, 2004), no significant upregulation of autophagy was observed in the present analysis.

In addition to phagocytosis, the catalytic function is also significant enriched, the upregulated proteins in this pathway such as corin protease was proved to play an essential role in the physiological catalytic function of bivalve. Corin protease is a family of alkaline proteases containing various hydrolytic enzymes such as trypsin and elastase. Researches have reported that upregulating expression of corin could promote hydrolysis of symbiotic bacteria, which is a critical nutritional strategy for bivalves in food-deprived environments (Herry et al., 1989; Boetius and Felbeck, 1995). Moreover, some immune-related proteins have also significantly expressed under starvation. One of them is the cell surface antigen CD109, which is an essential cellular immune protein that has been shown to function in immune in both human skin and *Euprymna scolopes* (Yazzie et al., 2015). Among them, the survival of *E. scolopes* with symbiotic bacterial communities leads to decreased CD109 expression levels and, thus, host immunity. It is hypothesized that significant downregulation of CD109 in mussels after starvation leads to a decrease in immunity. However, mussels in starvation also have remedies approaches to enhance immunity, such as elevated expression levels of CTSL, a vital member of the histone protease family (Chen et al., 2019). CTSL is involved in innate immune response and apoptosis in turbot bacterial infestation experiments, and it is also a histone enzyme in the late phase of cell phagocytosis. Presumably, CTSL is significantly upregulated to ensure mussels do not suffer from bacterial infection under prolonged starvation and participate in the apoptotic pathway. The upregulation of CTSL protein explains the ability of the mussel to fit prolonged starvation from an immune perspective.

The metabolism-related pathways are purine metabolism, pyrimidine metabolism, nicotinic acid and nicotinamide metabolism, and the KEGG enrichment analysis showed that some downregulated proteins were significantly enriched in all three pathways (e.g., NME5, DeoA, PunA). Purine metabolism refers to the synthesis and catabolism of purines and their derivatives in organisms, which play an essential role in energy supply. Pyrimidine metabolism includes the synthesis, degradation, recycling, and interconversion of DNA, RNA, lipids and carbohydrates. Purine and pyrimidine metabolism response to starvation have been documented in the previous literature. Alizadeh et al. (2021) found that the purine metabolism capacity of Russian sturgeon was reduced after prolonged starvation. Moreover, Xie et al. (2021) found that downregulated proteins in the purine and pyrimidine metabolism pathways were enriched in Australian jewel perch after starvation. The reduction of all three metabolic capacities is inseparable from reducing energy expenditure. In addition, the degradation and recycling of DNA, organelles, and cytoplasm are related to all three metabolic pathways. Notably, inhibition of purine metabolism, which is almost identical in eukaryotes, reduces unwanted cell proliferation (Kokina et al., 2019). It implies that mussels reduce their energy consumption by inhibiting their growth in a starved state, which may also lead to the cessation of reproduction in the reproductive stage, a phenomenon also presented in oysters (Huvet et al., 2010).

In addition to the reduced energy supply of the above pathways, the energy supply of mussel motile cells is also restricted. According to the GO functional annotation analysis, mussel cilia activity and motile cilia cell activity are significantly enriched in downregulated proteins (e.g., TEKT1). Furthermore, starvation has been shown to induce ciliated cell autophagy and inhibit primary cilia formation (Xie et al., 2022). The inhibition of primary cilia formation is also a sign of energy conservation. The primary function of motor cilia is to oscillate directionally to expel bacteria, dust and other materials from the surface of the cilia. The reduced motility of motor cilia cells may be due to the redistribution of energy and nutrients under prolonged starvation, where vital organs that sustain life can allocate far more energy and nutrients than other organs, such as motor organs, which include motor cilia cells. This situation is similar to the trade-off between metabolic energy consumption and energy storage in the cavefish, which means that only after enough energy has been stored to meet survival needs does the roseate loach allocate energy to visual growth. This physiological activity is not conducive to survival in caves (Zhao et al., 2019). Different proteomics and quantitative fluorescence PCR results showed downregulation of hepatocyte growth factor (HGF) and hypoxia-inducible factor (HIF-1). HGF is a hormone that stimulates hepatocyte proliferation, and HIF-1 is a nuclear protein with transcriptional activity. Both of which inhibit starvation-induced apoptosis (Tacchini et al., 2004; Gallo et al., 2014), and apoptosis on damaged cells clearance has been studied as a maintenance behavior towards the tissue (Meier et al., 2000), which is also closely related to the phenomenon of cilia lysis in electron microscopy results.

## 5 Conclusion

In the present study, a quantitative proteomics was performed in *M. coruscus* gill tissue under starvation stress, as well as the morphological change of gill filament was observed, to figure out the coping strategies of mussel under starvation. Our results revealed that a few phagosome-related proteins, like ATPeV1E, ATPeV1C, LAMP1 2 and CTSL were significantly upregulated after starvation (*p* < 0.05), in addition to this, some other pathways related to immunity and metabolism had also significantly enriched. The gill morphological observation showed an autolysis of gill cilia and widen gaps between gill filaments. The corin protease content in gill and MPO activity in blood both significantly increased and more dead cells were found in blood. Those results suggest that the coping strategies of mussels in response to starvation are a series of physiological activities and as well as various protein regulatory pathways, such as phagocytosis activation, immune maintenance and metabolic reduction.

## Data Availability

The datasets presented in this study can be found in online repositories. The names of the repository/repositories and accession number(s) can be found in the article/Supplementary Material.
