# Supplementary figures and images for: Morphological change and differential proteomics analysis of gill in Mytilus coruscus under starvation
Source: Front Physiol. 2023 Mar 30;14:1150521. doi: 10.3389/fphys.2023.1150521 (PMC10097965; doi:10.3389/fphys.2023.1150521)

A

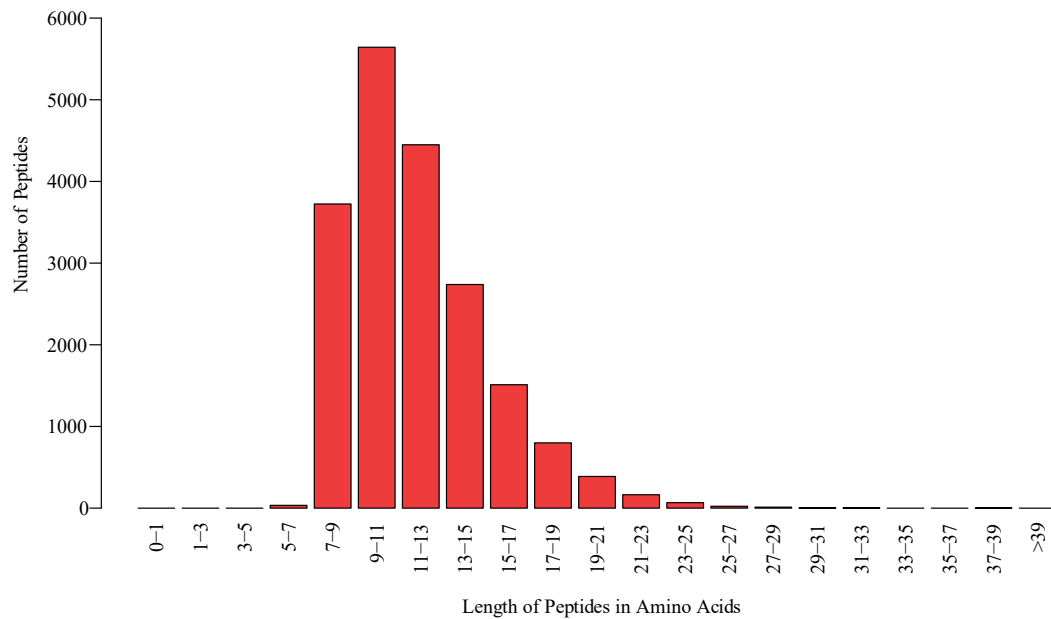

B

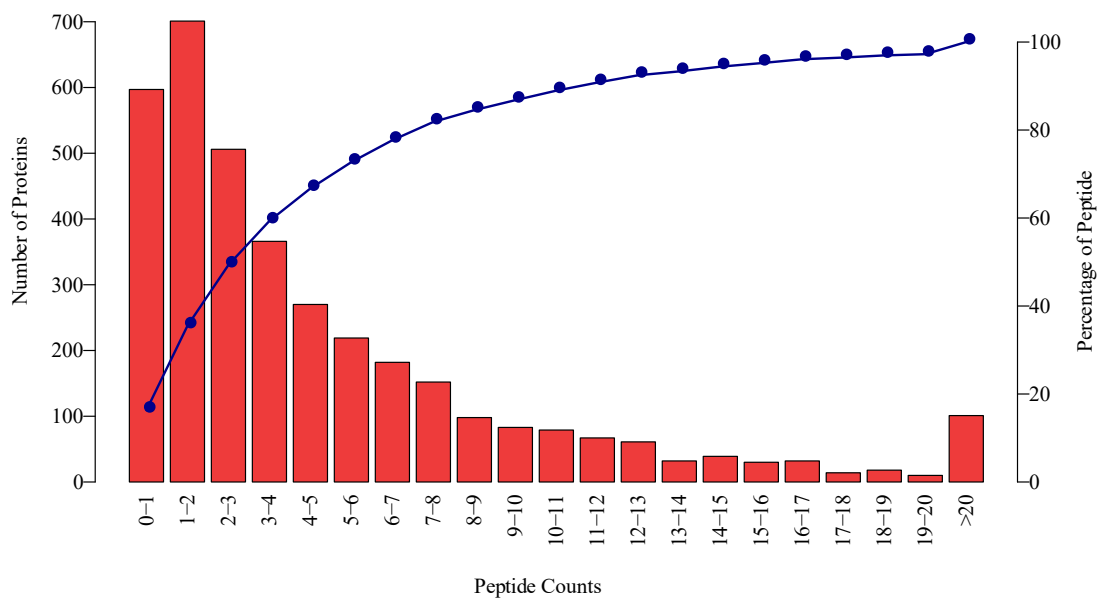

Supplement: Supplementary file 1 [file DataSheet1.PDF]
